# Quercetin and Bornyl Acetate Regulate T-Lymphocyte Subsets and INF-*γ*/IL-4 Ratio *In Utero* in Pregnant Mice

**DOI:** 10.1155/2011/745262

**Published:** 2010-09-29

**Authors:** Xiaodan Wang, Aituan Ma, Wanyu Shi, Meiying Geng, Xiuhui Zhong, Yantao Zhao

**Affiliations:** ^1^College of Traditional Chinese Veterinary Medicine, Agricultural University of Hebei, Baoding 071001, China; ^2^College of Animal Science and Veterinary Medicine, Agricultural University of Hebei, Baoding 071001, China

## Abstract

The objective of this study is to investigate the antiabortive effects of Quercetin and Bornvl Acetate and their immunological modulation at maternal-fetal interface. Lipopolysaccharide (LPS) was injected via tail vein to induce abortion in mice which received Quercetin and Bornvl Acetate at days 4–7 of gestation. Uterine CD4+/CD8+ T lymphocytes and IFN-*γ*/IL-4 of each group (*n* = 10) were detected by immunohistochemistry and enzyme-linked immunosorbent assay, respectively. The ratio of CD4+/CD8+ increased significantly (*P* < .01) in the uterus of LPS-induced abortion mice. In the Quercetin and Bornvl Acetate pretreated mice followed by LPS administration, the ratio of CD4+/CD8+ dropped to 0.562 ± 0.021, lower than that of LPS-abortion group (*P* < .01). The mean value of IFN-*γ*/IL-4 in LPS-treated mice was 0.310 ± 0.066, higher than that of Quercetin and Bornyl Acetate group. The results indicate that Quercetin and Bornyl Acetate have an antiabortive effect through modulation of immunological balance at maternal-fetal interface.

## 1. Introduction

Pregnancy is a complex, sophisticated physiological process. During this process, the embryo is viewed as a semiallograft due to the expression of paternal antigens that are foreign to the mother and a total allograft in fertilization* in vitro* and embryo transfer (IVF-ET). The semiallogenic nature of the fetus is not usually rejected by the maternal immune system, which includes complicated immune modulation. In recent years, the contribution of focalized immunity at the maternofetal interface to successful embryo implantation and growth has been studied to a great extent; maintaining the maternofetal tolerance is a main concern for reproductive immunologists. Cases of pregnancy failure, such as spontaneous abortion, have been studied for association with altered immune responses [1, 2]. In 1993, Wegmann et al. [3] first raised the hypothesis that there was a pregnancy-preferred Th2 bias involving the downregulation of the cellular immune response, and the upregulation of humoral immune response. There have been a great number of studies which support Wegmann's hypothesis [4, 5]. 

 T lymphocytes involved in gestation immunity account for the third most abundant leukocyte population in normal early pregnancy decidua, accounting for 20%–30% of stromal leukocytes. Endometrial T lymphocytes participate in the defense mechanism of uterus, and either secreting cytokines when stimulated or acting as local suppressor cells. The human decidua contains an unusually high proportion of lymphocytes, mainly NK and T cells, which are potentially cytotoxic to the trophoblast when they are stimulated with certain cytokines, resulting in embryo loss. Olivares et al. [6] reported that activated decidual lymphocytes participate in human spontaneous abortion by inducing apoptosis but not necrosis of the trophoblast. 

 Quercetin is an herbal flavonoid derived from various kinds of plants and has been found to possess various biological activities, such as antioxidative [7, 8], antimicrobial [9], wound-healing [10], anticancer [11, 12] and immunomodulatory activities [13]. In Chinese herbal medicine, Quercetin is the major constituent of many herbs, such as *Semen Cuscutae*, *Herba Taxilli*, *Cortex Eucommiae*. Bornyl Acetate constitutes the major components in the oil of *Fructus Amomi*. It is recorded in the Chinese Veterinary Pharmacopoeia (the 2005 edition) that *Semen Cusutae, Herba Taxilli*, *Cortex Eucommiae, *and *Fructus Amomi* are antiabortives. So far, there have been few reports on whether Quercetin and Bornyl Acetate are the effective components of the antiabortive herbs, and how these components affect the survival of the fetus. In the present study, Lipopolysaccharide (LPS) was administered by injection to pregnant mice to induce embryo resorption. Uterine CD4+ and CD8+ T cells were detected immunohistochemically, and IFN-*γ* and IL-4 contents were measured by Enzyme-linked immunosorbent assay (ELISA) with an aim to elucidate the antiabortive effects and the mechanisms of Quercein and Bornyl Acetate.

## 2. Materials and Methods

### 2.1. Preparation of Reagents

LPS (Lipopolysaccharide, Sigma Chemicals) from *Escherichia coli* was dissolved in sterile phosphate-buffered saline (PBS) (0.01 M, pH 7.4) at a concentration of 0.5 *μ*g/mL. Ten mg of Quercetin (Sigma products, purity = 98%) was dissolved with a small amount of dehydrated alcohol and then diluted with PBS to 4 mL, the final concentration being 2.5 mg/ml. An amount of 10 *μ*L Bornyl Acetate (Fluka products, purity ≥99%) was dissolved with small amount of dehydrated alcohol, and then diluted with PBS to 4 mL. Quercetin (5 mg) and Bornyl Acetate (5 *μ*L) was mixed and dissolved with a small amount of dehydrated alcohol, and then diluted with PBS to 4 mL.

### 2.2. Treatment of Animals

BALB/c virgin mice aged 10 weeks (18–22 g BW) were purchased from the Laboratory Animal Center of Hebei Medical University, China and were given free access to mouse chow and water, with a 12 hours light cycle from 7:00–19:00. Pregnancies were obtained by housing one virgin female with one male, and the females were examined each day in the early morning for the presence of a vaginal plug. The day that the vaginal plug was detected was designated as day 0 of pregnancy. 

 The pregnant mice were randomly divided into 5 groups. Group A was kept as a control group, group B as a LPS model group, group C as a Que (Quercetin) group, group D as a BA (Bornyl Acetate) group, and group E as a Que + BA group. Mice in groups B, C, D, and E were given intravenous injections (i.v.) of 0.2 mL (0.1 *μ*g) LPS/mice via the lateral tail vein on day 7. Mice in groups C, D, and E received Que, BA, and Que + BA respectively at a dose of 0.4 mL per day via oral gavage on gestation days 4–7. Animals in group A received an oral gavage of PBS on the same days of gestation as in group C, and were administrated with PBS at 0.4 mL at day 7 of gestation. Animals were treated according to the Guidelines for Keeping Experimental Animals issued by Chinese government.

### 2.3. Calculation of Embryo Loss Rate and Abortion Rate

All gravid females were sacrificed by cervical dislocation at day 9 of gestation and the contents of uterus were examined for viable and resorbing embryos. The viable embryos (V) were well-oxygenated (pink) and showed a well-defined embryonic capsule and placenta. The resorbing embryos (R) were usually smaller, showed signs of ischemia, haemorrhage, and often were macerated and black in color without identifiable embryo or placenta. The incidence of embryo loss was presented as a percentage of the contents of the uterus (100 · R/(V + R)). The incidence of abortion was calculated as a percentage of the contents of the miscarriage (100 · abortive mice/total mice).

### 2.4. ELISA Assay for IFN-*γ* and IL-4

All pregnant mice were sacrificed by cervical dislocation at day 9 of gestation. The left parts of uterine horns used for ELISA were carefully cleaned, with the fetuses removed. Uterine lysates were prepared in PBS (pH 7.4) containing PMSF (0.75 *μ*g/mL, phenylmethylsulfonyl fluoride, Thermo Scientific, IL, USA), and centrifuged for 15 minutes at 12000 r.p.m. at 4°C and the supernatants were collected for ELISA. IFN-*γ* and IL-4 levels were assessed using an IFN-*γ* ELISA kit (R&D Systems, USA) and an IL-4 ELISA kit(R&D Systems, USA) respectively according to the manufacturer' instructions.

### 2.5. Immunohistochemistry

The gravid females were sacrificed by cervical dislocation at day 9 of gestation. The uterine horns (right) were fixed in Bouin's fluid, dehydrated in graded ethanol, and embedded in Paraplast. Then serial sections of 6 *μ*m thick were prepared. Rat antimouse CD4+ monoclonal antibody (IgG2b, Serotec, UK), and rat antimouse CD8+ alpha monoclonal antibody (IgG2a, Serotec, UK) were employed in the study. The uterine sections were deparaphinized and hydrated, and stained using the streptavidin immunoperoxidase technique. Briefly, the tissue sections were overlayed with CD4+ or CD8+ alpha after citrate pretreatment, and incubated in a humid chamber at 4°C overnight. Then the sections were allowed to react with a biotinylated rabbit antirat IgG (H+L) antibody (Vector, UK) in 10% normal calf serum, followed by incubation with horseradish peroxidase conjugated streptavidin (Vector, UK). The tissue sections were washed thoroughly with PBS before each procedure. Finally, the sections were incubated with 0.03% diaminobenzidine DAB containing 0.05% hydrogen peroxide and then mounted.

T cells were measured under light microscope (magnification 40x for the objective lens). All positive cells in the field were counted. The mean value was pooled from twenty high-power fields in each sample, and was averaged from ten mice.

### 2.6. Statistical Analysis

Differences in T cells and IFN-*γ*/IL-4 contents were assessed by one-way analysis of variance. Statistical analysis of abortion rate and embryo resorption rate was conducted using *χ* ^2^-test (*P* < .05 was taken as significant).

## 3. Results

### 3.1. The Antiabortive Effects of Quercetin and Bornyl Acetate

The group A control mice pretreated with PBS showed a 20% natural abortion rate. The abortion rate in the group B mice was 100.0% and was comprised of an embryo resorption rate of 100.0%. The resorbed conceptus was severely macerated and black in color. Mice in group C, pretreated with Que, showed an abortion rate of 50.0% and a resorption of 53.2%. Mice in group D, pretreated with BA, showed an abortion rate of 60% and a resorption rate of 60.4%. Mice in group E, pretreated with Que and BA, showed an abortion rate of 30.0% and a resorption rate of 28.6% (Table 1).

### 3.2. Que and BA Alter Uterine IFN-*γ* and IL-4 Contents Either Used Alone or in Combination

**Figure 1:**
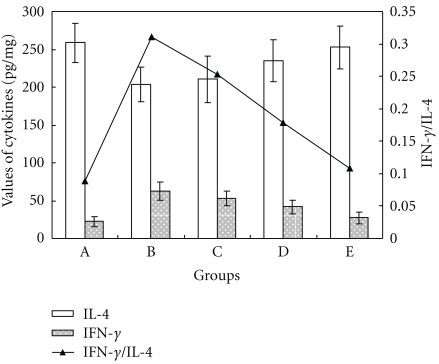
Changes of IFN-*γ*/IL-4 in uterus of mice (*n* = 10) in different groups. Values of IFN-*γ* and IL-4 were expressed as pg/mg protein. The figure axis showed the levels of IFN-*γ* and IL-4, and the secondary figure axis showed the ratio of IFN-*γ*/IL-4.

**Figure 2:**
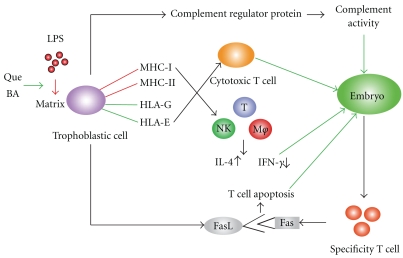
The effects of LPS on the increased expression of major histocompatibility complex (MHC) I and II, and the stimulus of T lymphocyte, natural killer cell (NK) and macrophage, bringing the level of IFN-*γ* up and the IL-4 down. Quercetin and Bornyl Acetate reverse the effects of LPS, and recover the immune balance at the maternal-fetal interface, thus protect the embryo.

All mice in group B treated with LPS (0.1 *μ*g/mice) aborted. Their INF-*γ* levels were increased (62.679 ± 12.362) compared with the PBS-treated mice in group A (22.311 ± 6.862), and their IL-4 levels were decreased from 258.572 ± 25.925 (group A) to 203.292 ± 22.815 (group B) (Figure 1). Consequently, the ratio of IFN-*γ*/IL-4 in group B was increased significantly compared with group A. After separate or combined Que or BA pretreatment (Groups C, D, and E), the IFN-*γ* levels were downgraded and IL-4 levels were upregulated in different degrees, and the ratios of IFN-*γ*/IL-4 were decreased significantly (*P* < .01) compared with group B (LPS treatment only). These results indicate that Que or BA alone has a protective effect on LPS-induced IFN-*γ*/IL-4 changes in the uterus and this effect is enhanced with combinatory Que and BA. See Figures 1 and 2.

### 3.3. Que and BA Influence the Population of Uterine CD4+/CD8+ Cells

**Figure 3:**
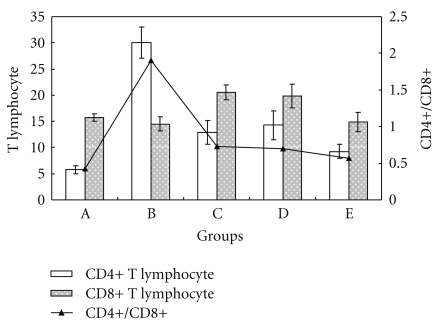
Changes of CD4+/CD8+ T lymphocytes in uterus of mice (*n* = 10) in different groups. The figure axis showed the amount of CD4+ and CD8+ T lymphocytes, and the secondary figure axis showed the ratio of CD4+/CD8+ T lymphocytes.

**Figure 4:**
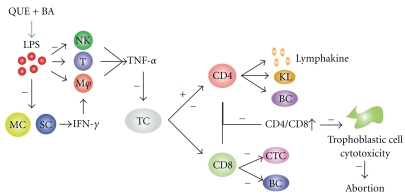
Differentiation of T lymphocyte is stimulated by LPS with the amount of CD4+ T lymphocyte increased, enhancing the immunological reaction of matrix and trophoblastic cell cytotoxicity. The experiment result indicates Quercetin and Bornyl Acetate can obstruct the effect of LPS. MC: mononuclear cell; SC: spleen cell; KL: killer T lymphocyte; CTC: cytotoxic T cell.

Both CD4+ and CD8+ T lymphocytes were seen in the uterus of control mice, and the CD8+ cells were less numerous than the CD4+ T lymphocytes. Mice treated with LPS in group B, had a higher uterine CD4+ T lymphocyte number, with no significant change in the CD8+ lymphocytes. When the mice were pretreated with Que and BA either alone or in combination, the greatest decrease in CD4+ cells was observed with combined Que and BA administration (group E). When Que or BA was administered alone (groups C and D), there was a significant increase in CD8+ T lymphocytes. However, there was no significant difference in the CD8+ T lymphocyte count when Que and BA were administered together (group E) compared to the control (group A) and LPS only (group B). See Figures 3 and 4.

**Figure 5:**
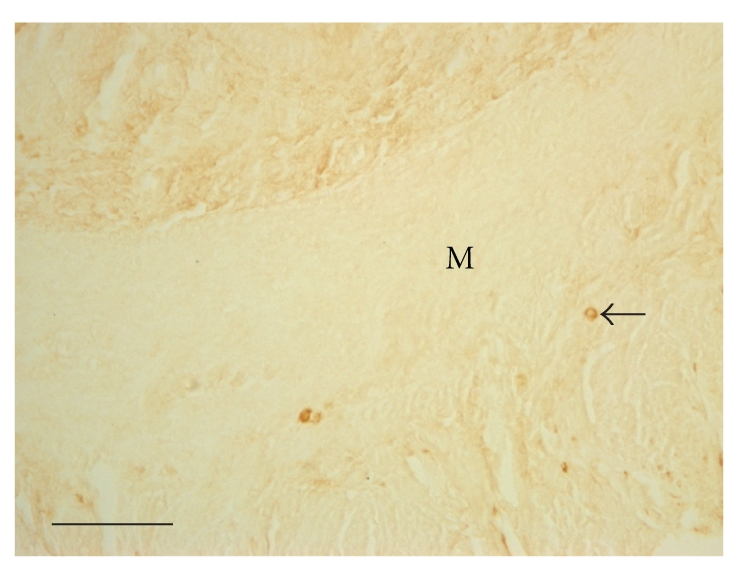
Lymphocytes (arrow) are most commonly concentrated in the myometrium in the control mice and are less in number. DAB stained. M, Myometrium. The bar shows 30 *μ*m.

**Figure 6:**
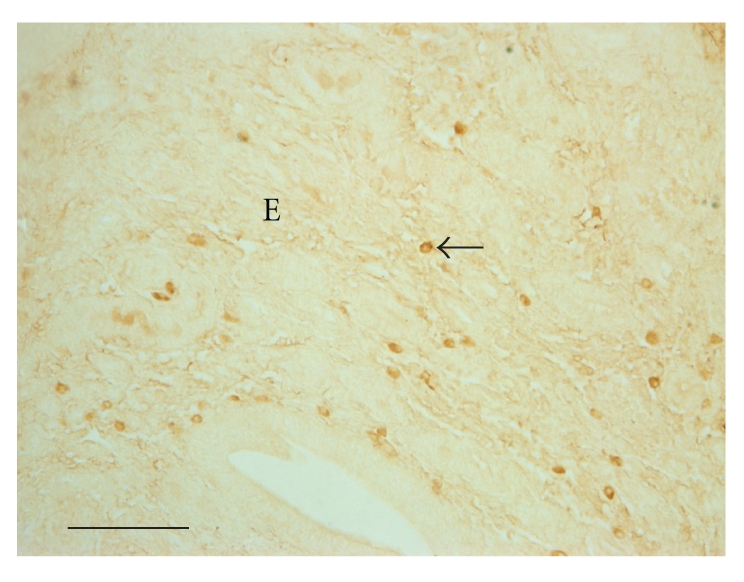
More lymphocytes (arrow) are present in the endometrium in the abortion groups. DAB stained. E, Endometrium. The bar shows 30 *μ*m.

 There is a larger presence of lymphocytes in the tissue of aborted mice. The lymphocytes are most commonly concentrated in the myometrium in the control or Que and BA-treated mice as shown in Figure 5, or in the endometrium in abortion groups as shown in Figure 6.

## 4. Discussion

Previous studies showed that exposure to *Escherichia coli *endotoxins contributed to the termination of pregnancy at any stage of gestation [14, 15], but little is known about the mechanisms involved. The possible mechanisms of LPS-induced abortion might be due to the secretion of tumor necrosis factor-alpha (TNF-*α*) by triggered macrophages (M*φ*), which act on placental blood vessels directly, resulting in hemorrhagic necrosis [16]. Priming of macrophages from both murine and human sources by recombinant immune interferons from *Escherichia coli* (IFN-*γ*) and activation by lipopolysaccharide (LPS) resulted in the production of tumor necrosis factor [17]. LPS, that predominantly induces TNF-alpha/beta in fresh murine splenocytes, is able to stimulate T lymphocytes to produce interferon-gamma (IFN-*γ*) [18]. Treatment of pregnant mice during early gestation with 0.1 *μ*g LPS resulted in 100% fetoplacental resorption on day 7 of gestation [19], and the resorption induced by LPS was significantly reduced by pretreatment with a TNF-*α* antagonist pentoxifilline (PXF), suggesting that TNF-*α* was involved in LPS-induced fetal resorption [20]. Terranova and Montgomery Rice [21] reported that endotoxin inhibits gonadotropin-stimulated ovarian steroidogenesis and follicular development and these effects were mediated by direct effects of LPS on ovarian cells, and in part, by TNF-*α*. In addition, nitric oxide (NO) fulfils important functions during pregnancy and has a role in implantation, decidualization, vasodilatation and myometrial relaxation. However, at high concentrations, such as those produced in sepsis, NO has toxic effects as it is a free radical. LPS induced 100% embryonic resorption at 24 hours post injection, with complete fetus expulsions at 48 hours. These results show that NO fulfils a fundamental role in LPS-induced embryonic resorption [22]. In the present study, both abortion rate and embryo resorption rate reached 100% (*P* < .01) after an intravenous injection of LPS via the lateral tail vein at a dose of 0.2 mL (0.1 *μ*g) on day 7. 

 Quercetin is a constituent of Semen Cuscutae, Herba Taxilli, Cortex Eucommiae and so forth. and Bornyl Acetate is a constituent of Fructus amomi. Modern pharmacological research has shown that the Quercetin is a major active component of Semen cuscutae and has comprehensive biological action. Lin and others [23] reported that Quercetin protects TNF-*α*-induced injury of cultured vascular endothelial cells (VEC)-304 and its mechanism of action may be related to the decrease of NO, and it's antioxidant effect. The antioxidant effect might be exerted through the nuclear factor-kappa B (NF-kappa B) activation pathway. Researches have shown that the overproduction of TNF-alpha and NO by LPS-stimulated macrophages is markedly inhibited by quercetin [24]. Liang et al. [25] observed that quercetin can inhibit the adhesion of platelets to human umbilical vein endothelial cells induced by TNF-alpha and the expression of adhesion molecules (P-selectin and ICAM-1). Bornyl Acetate has the actions of antidiarrhea, analgesia, dephlogistication, depressing spasm. So far, it has not been reported whether Quercetin and Bornyl Acetate have an immunoregulatory effect on pregnant mice. In the present study, LPS induced embryo resorption in mice, while when pretreated with Quercetin and Bornyl Acetate either alone or in combination, the abortion rate was decreased significantly (see Table 1). The effect of Quercetin combined with Bornyl Acetate on LPS-induced abortion was even more significant than either Quercetin or Bornyl Acetate alone; abortion was decreased compared to that of the LPS-induced-abortion group (*P* < .01). Thus, the present study demonstrates that Quercetin and Bornyl Acetate have an antiabortive effect in mice.

 Immunological mechanisms have been suggested to contribute to early pregnancy loss. The imbalance of T-lymphocyte subsets in the decidua is associated with recurrent spontaneous abortion (RSA). Previous study [26] proved that the percentage of CD4+ T cells in the decidua of RSA patients was higher than that in the controls (*P* < .05), while there was no significant difference in CD8+ T cells between the two groups (*P* < .05). CD8+ T cells have been proposed to have an immunoregulatory role in normal pregnancy. Defciency in suppressor cells and factors has been suggested to contribute to the pathogenesis of spontaneous abortion [27, 28]. However, Vassiliadou had demonstrated that in common with normal pregnancy, most endometrial T cells expressed CD8+, thus implying that reduced CD8-positive cell-mediated immunosuppression is unlikely to play a role in adverse pregnancy outcome [29]. CD4+ T lymphocytes include T-helper (Th) 1 and Th2 cell subpopulations. Th1 cells secrete the T-helper 1 cytokines, such as IL-2, TNF-*α*, interferon-*γ*, which have been shown to have detrimental effects on embryo development and trophoblast growth *in vitro* [30, 31]. Th2 cells produce a range of T-helper 2 cytokines, such as interleukin (IL)-4, IL-6 and IL-10, and it has been postulated that during normal pregnancy T-helper 2 cytokines, which control the humoral arm of the immune response, are benefcial and preferentially secreted [3]. In the present study, immunohistochemistry analysis showed that uterine CD4-positive lymphocytes were increased in number with LPS-induced abortion compared to that of normal pregnancy. In other words, the ratio of CD4+/CD8+ was increased in LPS-induced abortion mice compared to that of normal mice in accordance with previous findings [32]. When Quercetin and Bornyl Acetate were administered in combination to prevent LPS-induced abortion, less CD4+ T cells were counted in the uterine tissue. When LPS-induced mice were administered Quercetin with Bornyl Acetate, the ratio of CD4+/CD8+ was significantly decreased from 1.907 ± 0.06 to 0.562 ± 0.021 (*P* < .01) and was comparable to the PBS-administered control mice value (0.429 ± 0.012). 

 A great number of studies indicate that a Th2 cytokines profile may be associated with normal pregnancy, whereas the lack of a dominant Th2 cytokines profile may be indicative of a pathologic pregnancy [33]. Significantly higher concentrations of Th1 cytokines were produced in women with recurrent spontaneous abortion as compared to women in their first trimester normal pregnancy, indicating a distinct Th2-bias in normal pregnancy and a Th1-bias in unexplained recurrent spontaneous abortion [31, 34]. IFN-*γ* primes and triggers decidual macrophages and leads to early embryo losses [35]. Peripheral blood lymphocytes from women suffering unexplained recurrent spontaneous abortion produce the T-helper 1 cytokine interferon-*γ* which has been shown to have detrimental effects on embryo development and trophoblast growth *in vitro* [30, 31]. IL-4 is secreted by Th2 cells, which are the major modulating cells of humoral immunity. IL-4 can promote the proliferation of B cells. It also can regulate the Th1/Th2 cytokine balance [36] and it is now accepted that changes in the balance of Th1/Th2 type cytokines occur during pregnancy in the feto-placental unit. These changes contribute to the implantation of the embryo, development of the placenta and survival of the fetus to term [37]. In the current study, high levels of Th1 type cytokine IFN-*γ* were found in LPS-induced abortion mice, while the Th2 type cytokine IL-4 levels were decreased significantly when injected with LPS on day 7 of pregnancy. It is suggested that the mechanism of LPS induced embryo loss might be through inducing a Th1 shift in the Th1/Th2 immune balance, resulting in abortion. 

 In conclusion, our study provides evidence that alterations in both the CD4+/CD8+ and IFN-*γ*/IL-4 ratios participate in LPS-induced fetal resorption, and that the Quercetin and Bornyl Acetate have an antiabortive effect via maintenance of the CD4+/CD8+ T lymphocytes and IFN-*γ*/IL-4 balance in uterus. Future studies will focus on the systematic investigation of the expression of activation markers by decidual leukocytes and on functional analysis of well-defned highly purifed populations.

## Figures and Tables

**Table 1:** Gestation results of different treatments (*n* = 10).

Group	Drug or PBS i.g. (mL)	LPS or PBS i.v. (mL)	Abortion rate (%)	Rate of resorption (%)
	(days 4–7 of gestation)	(day 7 of gestation)		
A	PBS 0.4	PBS 0.2	20.0 (2/10)**	24.4**
B	PBS 0.4	LPS 0.2	100.0 (10/10)	100.0
C	Que 0.4	LPS 0.2	50.0 (5/10)*	53.2**
D	BA 0.4	LPS 0.2	60.0 (6/10)*	60.4*
E	Que + BA 0.4	LPS 0.2	30.0 (3/10)**	28.6**

Notes: Comparison of abortion rate and fetal resorption, *P < .05 and **P < .01 compared with group B.
